# Successful reduction of inflammatory responses and arachidonic acid–cyclooxygenase 2 pathway in human pulmonary artery endothelial cells by silencing adipocyte fatty acid-binding protein

**DOI:** 10.1186/s12950-017-0155-6

**Published:** 2017-03-20

**Authors:** Qian Wang, Guanglin Shi, Ying Teng, Xia Li, Jin Xie, Qin Shen, Caixin Zhang, Songshi Ni, Zhiyuan Tang

**Affiliations:** Department of Respiratory Medicine, Affiliated Hospital of Nantong University, Nantong, 226001 Jiangsu China

**Keywords:** FABP4, Inflammation, AA, COX2, PTE

## Abstract

**Background:**

Adipocyte fatty acid-binding protein, also known as aP2 or fatty acid-binding protein 4 (FABP4), plays an important role in inflammatory and metabolic responses in adipocytes and macrophages. Recent work has demonstrated that macrophage FABP4 integrates inflammatory and lipid metabolic responses, thereby contributing to the development of insulin resistance and atherosclerosis. However, it is not known whether FABP4 in human pulmonary artery endothelial cells(HPAECs) modulates inflammation.

**Results:**

Here, we demonstrate that FABP4 and inflammatory cytokines are upregulated in lipopolysaccharide(LPS)-stimulated HPAECs. In addition, LPS increases the expression of molecules in the arachidonic acid(AA)–cyclooxygenase (COX) 2 signaling pathway in FABP4-expressing, but not FABP4-deficient, HPAECs.

**Conclusions:**

Our findings demonstrate that silencing FABP4 could decrease inflammatory cytokines, which were reported to be expressed via the AA–COX2 pathway, in HPAECs. In addition, silencing FABP4 could inhibit the expression of molecules in the AA–COX2 pathways. So we speculate silencing FABP4 could decrease the inflammatory response in HPAECs, which involves in the AA–COX2 signaling pathway. Our study suggests that FABP4 could be a potential biomarker and intervention point for the inflammation-related disease in HPAECs such as pulmonary thromboembolism.

## Background

Vascular endothelial cells inhibit platelet adhesion and anticoagulation in normal conditions, which is mainly dependent on the integrity of endothelial cells. Vascular endothelial injury is the most important and common cause of thrombosis [1]. And the inflammation, which might be the primary process or response to injury of endothelial cell, could lead to initiate thrombus formation [2]. Accompanied by pulmonary artery endothelial injury and inflammatory reaction, pulmonary thromboembolism(PTE) would initiate and develop [3, 4]. PTE is a common illness, which caused by the obstruction of thrombus in pulmonary artery and its branches, with high mortality and morbidity [5]. The most common diagnostic tests for PTE are the D-dimer blood test, which has poor specificity, and computed tomography, which has low sensitivity at the early stage of PTE [6]. Thus, there is an urgent need to identify accurate biomarkers to facilitate the early diagnosis of PTE.

Our previous studies showed that the expression of FABP4 is increased in an animal model of PTE [7]. FABP4 is a key enzyme in the regulation of intracellular lipid metabolism and, in turn, lipid metabolism is linked to inflammatory response [8]. Since the occurrence of PTE is accompanied by injury to vascular endothelial cells and inflammation, we postulated that FABP4 may involve in PTE-associated inflammatory activity.

FABP4 belongs to a family of small molecular weight (15 kDa) cytoplasmic fatty acid-binding proteins that bind with high affinity to unsaturated long-chain fatty acids [9]. FABP4 is expressed in a highly tissue-specific manner. In adipocytes, FABP4 plays a role in regulating lipid metabolism and insulin sensitivity. In macrophages, FABP4 regulates cholesterol ester accumulation and inflammatory activity [10]. A recent study has demonstrated that suppression of FABP4 protects mice against atherosclerosis and compromises the inflammatory responses of macrophages [11]. Another study showed that FABP4-deficient mice are resistant to several other inflammatory disorders [12]. These findings suggest that FABP4 modulates the inflammatory response in adipocytes and macrophages. However, the role of FABP4 in HPAECs has not yet been studied.

To address the question, we investigated the events underling LPS-stimulated FABP4 expression in HPAECs. Our study showed that PTE increases serum concentrations of the inflammatory mediators tumor necrosis factor-α (TNF-α), interleukin (IL)-1β, and IL-6. Since these mediators are induced downstream of AA metabolism by COX2 [13, 14], this finding suggested that the AA–COX2 pathway participates in PTE-associated inflammatory activity. AA is an unsaturated long-chain fatty acid that can be metabolized to diverse products depending on the expression and activity of various enzymes. In the normal physiological state, AA is stored in the cell membrane in the form of phospholipids, and released from the cell membrane when cells are activated by stimuli [15]. There were no reported of the relationship between FABP4 and the metabolism of AA, except a reported of AA could induce the expression of FABP4 in adipocyte [16]. So, the objective of the present study was to investigate the relationship between FABP4 and the AA metabolic cascade in inflammatory responses.

## Methods

### Human serum measurements

All subjects (20 PTE patients from Affiliated Hospital of Nantong University and 20 healthy volunteers) were fasted overnight for 12 h. The next day at 7:00, we extracted elbow vein blood placed in EDTA-anticoagulant tube, and then centrifuged it at 4 °C to separate serum. Cytokines were measured in serum by a commercially available ELISA kit (R&D System, USA). The study was approved by the Ethics Committee of Hospital Affiliated to Nantong University (2105-036).

### Cell culture

Human pulmonary artery endothelial cell (American Type Culture Collection, Manassas, VA, USA) were cultured in DMEM medium(HyClone, Logan City, Utah, USA) supplemented with 2 mM L-glutamine, 100 U/Ml penicillin, 100 mg/mL streptomycin and 10% fetal bovine serum (Gibco BRL, Grand Island, NY, USA). Cells were grown in a 5% CO_2_ humidified atmosphere at 37 °C.

### Cellular inflammation modeling

Cells were incubated in DMEM medium containing free bovine serum albumin overnight. They were then rinsed with serum-free DMEM medium and exposed to LPS from E.coli (SO55:B5; Sigma-Aldrich) or vehicle. We selected the most effective concentration and time by western blot and RT-PCR analysis.

### ShRNA transfection

Three small hairpin RNA targeting human FABP4 mRNA (named shRNA) and negative control duplex (named NC) were chemically synthesized by Biomics Biotech, Nantong, China. The sequences of shFABP4 and negative control shRNA were as follows: shRNA-1 sense,5’-GCAUGGCCAAACCUAACAUTdT-3’ and antisense,5’-AUGUUAGGUUUGGCCAUGCdTdT-3’; shRNA-2 sense,5’-CACGAGAGAUUUAUGAGAdTdT-3’and antisense,5’-UCUCUCAUAAACUCUCGUGdTdT-3’; shRNA-3 sense,5’-GGGAACCUUUCCACACUAUTT-3’ and antisense,5’-AUAGUGUGGAAAGGUUCCCTT-3’. Negative control shRNA sequences were (scramble) sense,5’-UUCUCCGAACGUGUCACGUTT-3’ and antisense,5’-ACGUGACACGUUCGGAGAATT-3’. Cells were then transfected by Lipofectamine 2000 reagents (Invitrogen, USA) according to the manufacturer’s instructions. We selected the one most effective silencing sequence by western blot and RT-PCR analysis.

### Cell viability

Cells were seeded on 96-well plates at a cell density of 1 × 10^5^ cells/mL. Cell viability was measured at different hours(0, 24, 48, 72 and 96 h) after transfected at shRNA concentration of 50 nM. 10ul CCK8 (Dojindo Laboratories, Japan) solution was added to the well. After incubated at 37 °C for 1 h, the cell absorbance (A) at 450 nm was measured at the MRX II absorbance reader (Dynex Technologies, USA). [Experimental group cell viability = (experimental group A value—cell free group A value)/(control group A value—cell free group A value) × 100%]. The experiment was repeated three times with 3 wells per group

### PCR (qRT-PCR)

Total RNA was extracted from cells using Trizol reagent (Invitrogen, Carlsbad, CA, USA), and reverse transcribed to cDNA using a Revert AidTM First Strand cDNA synthesis kit (Fermentas, Glen Burnie, MD, USA) following the supplier’s instructions. The primers (Table 1) used for real-time RT-PCR purchased from Biomics Biotech (Nantong, China). The transcripts were quantified with SyberGreen on an ABI 7500 thermal cycler (Applied Biosystems). The levels of expressed genes were quantified with the 2^-∆∆Ct^ method after normalizing to an endogenous reference GAPDH. The experiment was performed in triplicate.

**Table 1:** Primer sequences for real-time PCRs

Gene	Primer sequences (5’ → 3’)
GAPDH	ForwardGGTAGACAAGTTTCCCTT
	ReverseATATGTTCTGGATGATTCT
FABP4	ForwardGAATGCGTCATGAAAGGCG
	ReverseCAATGCGAACTTCAGTCCAGG
COX2	ForwardCTTTGACACCCAAGGGAGTC
	ReverseATCCTTGCTGTTCCCACCCA
IL-1β	ForwardAAACCTCTTCGAGGCACAAG
	ReverseGTTTAGGGCCATCAGCTTCA
IL-6	ForwardTCTCCACAAGCGCCTTCG
	ReverseCTCAGGGCTGAGATGCCG
TNF-α	ForwardCCTTCTCCAGCTGGAGAGC
	ReverseCGAGTGACAAGCCTGTAGC

### Western blot

The total proteins extract from HPAECs and were obtained using a lysis buffer (Beyotime Institute of Biotechnology, Nantong, China). Proteins were separated by SDS-polyacrylamidegel electrophoresis in 15% gels, and transferred to Polyvinylidene Fluoride(PVDF) Membrane (Millipore Corporation, USA) at 300 mA for 40 min. The membrane was blocked with 5% fat-free milk in PBS and 0.5% Tween-20 (TBST) for 1 h at room temperature(RT) and then incubated with the following primary antibodies overnight at 4 °C: monoclonal rabbit anti-FABP4 (1:500 dilution; Abcam, UK), monoclonal rabbit anti-COX2 (1:1000 dilution; Abcam, UK). After washing three times with TBST, horseradish peroxidase-conjugated (HRP)-conjugated goatanti-rabbit antibody (1:3000, biosharp, USA) was incubated with membrane for 1 h at RT. After stripping, the membrane was reprobed with β-actin(1:1000, Abcam, UK) overnight at 4 °C, followed by incubation with secondary antibody as above at room temperature for 2 h. Protein detection was performed using the enhanced chemiluminescence (ECL) system (Millipore, Bedford, MA).

### ELISA

Supernatants from cells (FABP4-deficient HPAECs or normal cells) were evaluated for secreted inflammatory cytokines, including TNF-α, IL-6 and IL-1β, by ELISA (R&D System, USA) after 0 or 1 μg/ml LPS treatment for 24 h. These cytokines were measured according to the manufacturer’s instructions. For analysis of PGE2 secretion, cells were also stimulated with LPS, and assayed via ELISA.

### LC-MS/MS determination of arachidonic acid

Arachidonic acid was quantitatively spiked into cell solution, and then takes the supernatants after super-speed centrifuged. Samples were separated on a Phenomenex Luna C_18_ column with a mobile phase, which consisted of water-0.02% formic acid (solvent A) and acetonitrile-0.02% formic acid (solvent B). A gradient separation was programmed at a flow rate of 200 μL/min-1 and temperature of the column was 35 °C. The electrospray ionization (ESI) mass spectrometer and negative and positive ionization modes mode to detect arachidonic acid (m/z 303.2) by liquid chromatography-tandem mass spectrometry (LC-MS/MS, AB SCIEX, Comcord, ON). An ion spray voltage was set to -5.5/4.5 kV (negative/positive) with electrospray ion source (turbo spray) temperature at 400 °C. All instrumentations were synchronized with Analyst Software (versions 1.5.1) of ABI.

### Statistical analysis

Data in the paper are expressed as means ± standard deviations (SDs) or means ± standard errors of means (SEMs). Statistical significance was determined by a one-way analysis of variance or Student’s *t* test.

## Results

### FABP4 expression is associated with the inflammatory response and is increased in HPAECs by LPS treatment

**Fig. 1:**
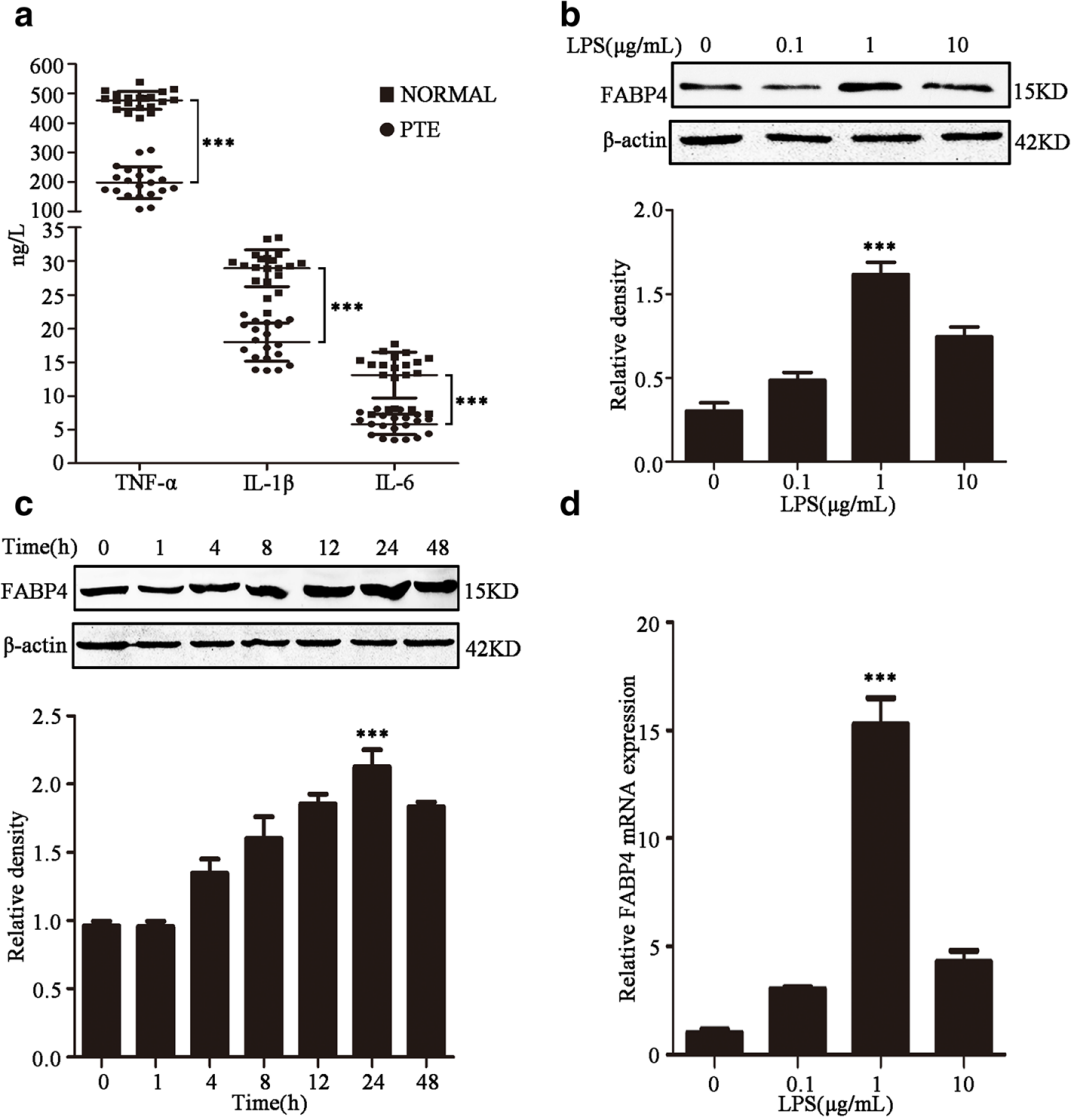
FABP4 expression is associated with the inflammation and is increased in HPAECs by LPS treatment. **a** The expression of TNF-α, IL-1β, IL-6 in the patients with pulmonary embolism and normal people were analyzed by ELISA. Results are shown as mean ± SD, *n* = 20, ***P < 0.001 compared to normal people. Western bolt was used to detect the protein level of FABP4 in cells stimulated with **b**) 0 μg/ml, 0.1 μg/ml, 1 μg/ml, 10 μg/ml of LPS and **c**) 1 μg/ml of LPS for 0 h, 2 h, 4 h, 8 h, 12 h, 24 h, 48 h. β-actin was used as loading control to quantitative analysis of the relative density. Data are presented as mean ± SD, *n* = 3, ***P < 0.001. **d** qRT-PCR was used to detect FABP4 mRNA expression in cells stimulated with four different concentrations of LPS normalized to GAPDH. Data are presented as means ± SEM, *n* = 3, ***P < 0.001

We found that levels of TNF-α, IL-1β, and IL-6 are significantly higher in the serum of patients with PTE compared with healthy subjects (*n* = 20) (Fig. 1a), as measured by ELISA, and these inflammatory cytokines were reported to be expressed via the AA–COX2 pathway [13, 14]. Compared with normal people, patients with PTE showed greatly increased secretion of IL-1β (28.95 ± 2.70 vs 18.01 ± 2.83 ng/L; *t* = 12.49, *P* < 0.001), IL-6 (13.11 ± 3.39 vs 5.79 ± 1.53 ng/L; *t* = 8.78, *P* < 0.001), and TNF-α (476.80 ± 30.05 vs 197.80 ± 53.73 ng/L; *t* = 20.27, *P* < 0.001). Using genomic and metabolomic approaches, we also found that FABP4 is upregulated in animal models of PTE. Thus, we speculate that the inflammatory response observed in PTE patients may be mediated by the AA signaling cascade. We first examined the expression of FABP4 in HPAECs. Western blot analysis showed that treatment of HPAECs with LPS significantly increased expression of FABP4 protein in a concentration- and time-dependent manner (Fig. 1b and c). Gray value analysis showed the protein level of FABP4 was highest in cells stimulated with 1 μg/ml(*F* = 111.12, *P* < 0.001) of LPS for 24 h(*F* = 70.29, *P* < 0.001). Consistent with the changes in FABP4 protein levels, FABP4 mRNA levels were elevated approximately 3-, 15- and 4-fold following stimulation with LPS at 0.1, 1, and 10 μg/ml LPS, respectively, for 24 h. Thus, the expression of FABP4 mRNA was highest in cells stimulated with 1 μg/ml LPS(*F* = 177.80, *P* < 0.001) (Fig. 1d).

### FABP4 expression is silenced in HPAECs transfected with FABP4-specific shRNA

**Fig. 2:**
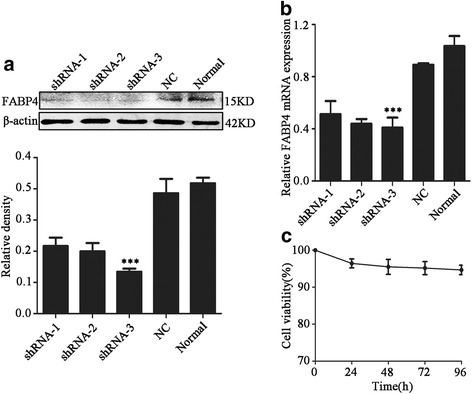
Three different sequences of shRNA for silencing FABP4 transfect into HPAECs. **a** Western blot was used to detect the protein level of FABP4 in normal cells and cells transfected by three different sequences of shRNA and negative control duplex(named NC). β-actin was used as loading control to quantitative analysis of the relative density. Data are presented as means ± SD, *n* = 3, ***P < 0.001. **b** qRT-PCR was used to detect the most effective sequence. Data are presented as means ± SEM, *n* = 3, ***P < 0.001. **c** CCK8 was used to detect the cell viability. Data are presented as means ± SEM, *n* = 3

We next tested three shRNA sequences for their ability to silence FABP4 expression in HPAECs by performing western blot and qPCR analysis of FABP4 protein and mRNA levels, respectively. Cells were transfected with one of three shFABP4 (shRNA-1, shRNA-2, and shRNA-3) or a negative control shRNA. We found that shRNA-3 was the most effective sequence for silencing FABP4 expression in both protein level(*F* = 57.79, *P* < 0.001) and mRNA level(*F* = 34.85, *P* < 0.001) (Fig. 2a and b), and this was therefore used in the following loss-of-function experiments. Then we examined the effect of shRNA transfection on cell viability at 0, 24, 48, 72 and 96 h (Fig. 2c). CCK8 assay showed there was no significant difference (*F* = 2.92, *P* = 0.077) in cell viability at all times. Thus, the damage of shRNA transfection to the cells could be negligible.

### LPS-induced expression of the inflammatory cytokines (TNF-α, IL-1β, and IL-6) is reduced in shFABP4 HPAECs

**Fig. 3:**
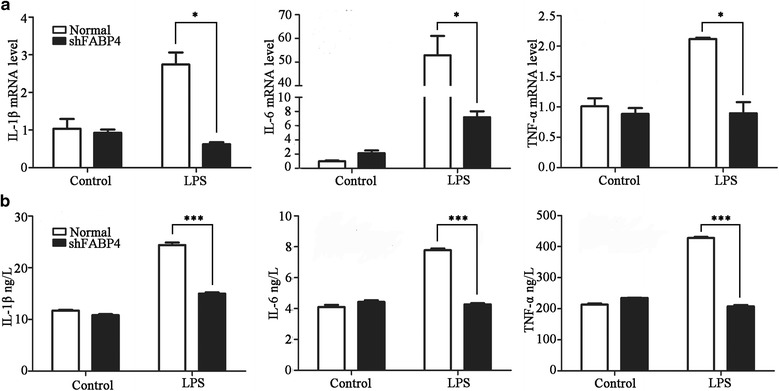
LPS-induced expression of inflammatory cytokines (TNF-α, IL-1β, IL-6) is weakened in shFABP4 HPAECs. TNF-α, IL-1β, IL-6 were produced from shFABP4-transfected cells and normal cells stimulating by LPS. **a** qRT-PCR and **b** ELISA were used to examine the expression levels of TNF-α, IL-1β, IL-6. Data are presented as means ± SEM, *n* = 3, *P < 0.05, ***P < 0.001

FABP4 participates in the inflammatory response evoked by various stimuli [17]. In the HPAECs (the untransfected and unstimulated cell), expression of the inflammatory cytokines (TNF-α, IL-1β, and IL-6) was increased in response to LPS stimulation, as detected by ELISA or qRT-PCR (Fig. 3a and b). To determine whether inhibition of FABP4 modulates the development of this inflammatory response, we transfected cells with shFABP4 and analyzed inflammatory cytokine mRNA levels in LPS-stimulated cells by qRT-PCR. This analysis showed that in LPS-stimulated group the cells transfected with shFABP4 expressed lower levels of TNF-α, IL-1β, and IL-6 mRNA(respective *t* = 6.62, *P* = 0.022; *t* = 6.82, *P* = 0.020 and *t* = 5.56, *P* = 0.031) compared with normal cells (Fig. 3a). We also examined cytokine protein secretion by ELISA. Compared with normal cells, shFABP4-transfected cells showed greatly reduced secretion of IL-1β (14.98 ± 0.39 vs 24.38 ± 0.83 ng/L; *t* = 17.71, *P* < 0.001), IL-6 (4.27 ± 0.13 vs 7.78 ± 0.18 ng/L; *t* = 26.82, *P* < 0.001), and TNF-α (207.39 ± 7.43 vs 427.63 ± 5.69 ng/L; *t* = 40.76, *P* < 0.001) in response to LPS stimulation (Fig. 3b). Thus, FABP4 deficiency attenuates the LPS-induced inflammatory response of HPAECs.

### FABP4 plays an important role in AA signaling cascade

**Fig. 4:**
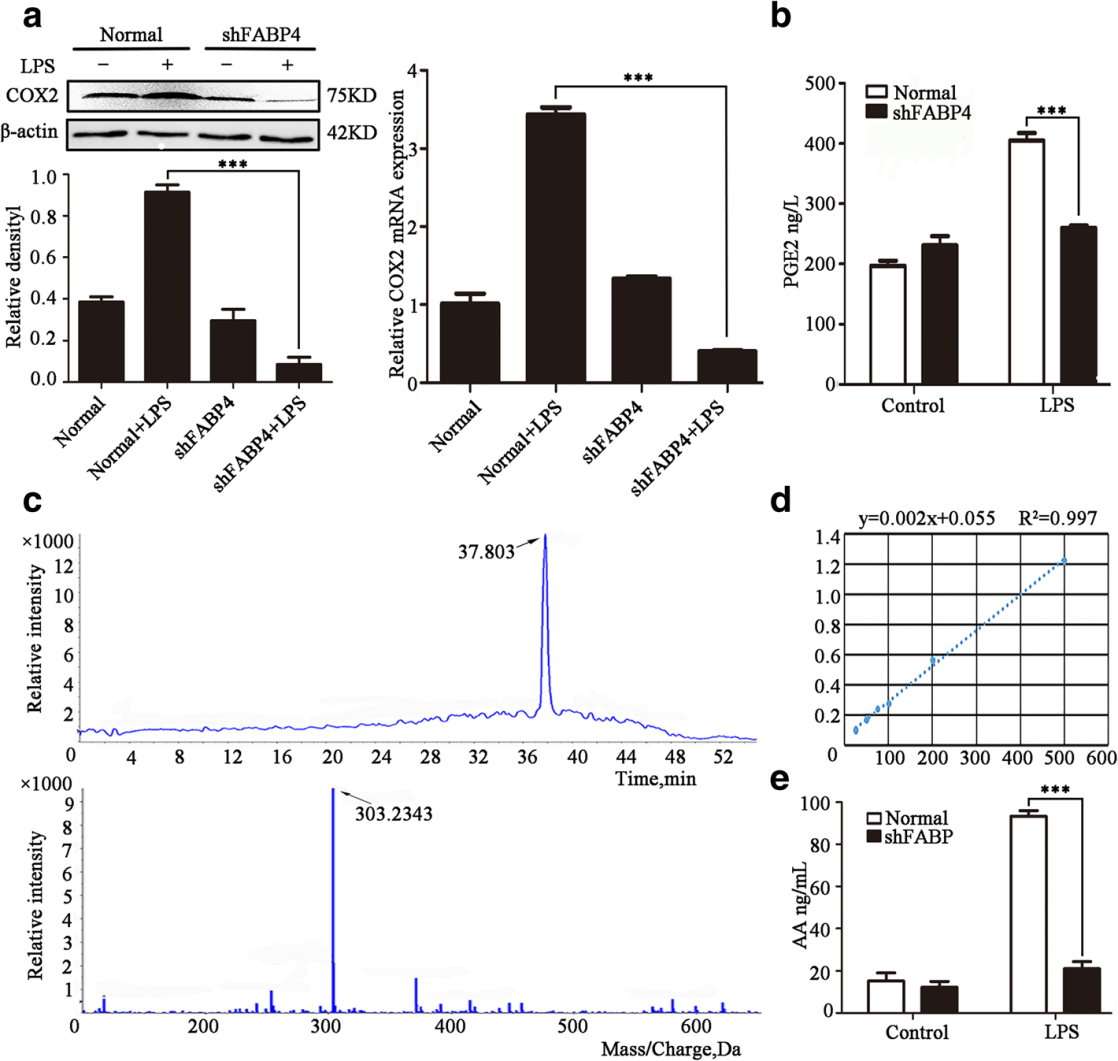
The expression of molecules related to AA signaling cascade were down-regulated in shFABP4 HPAECs. **a** The protein and mRNA level of COX2 were detected by western bolt and qRT-PCR. *n* = 3, ***P < 0.001. **b** ELISA was used to examine the expression levels of PGE2. Data are presented as means ± SEM, *n* = 3, ***P < 0.001. **c** Relative intensity of AA by means of LC and MS/MS. **d** Standard curve line of analytical standards of AA. **e** The levels of AA were detected in silencing FABP4 HPAECs and normal cells stimulating by LPS. Data are presented as means ± SD, *n* = 3, ***P < 0.001

We next investigated whether FABP4 is involved in AA signaling. Prostaglandin E2(PGE2), a product of AA metabolism by COX, is an important mediator of the inflammatory response. Stimulated with LPS, shFABP4-transfected cells showed greatly lower expression of COX2 in protein level(*t* = 27.85, *P* < 0.001) and mRNA level(*t* = 34.37, *P* < 0.001) than normal cells(Fig. 4a). Similar results were obtained when PGE2 levels were examined(Fig. 4b). Whereas there was no significant difference in PGE2 expression between unstimulated control(*t* = 1.97, *P* = 0.120) and FABP4-silenced cells(*t* = 1.86, *P* = 0.136), PGE2 expression in LPS-treated shFABP4-transfected cells was significantly lower than in stimulated control cells(*t* = 11.29, *P* < 0.001). We also analyzed AA levels in HPAECs by liquid chromatography-tandem mass spectrometry (LC-MS/MS) (Fig. 4c–e). As shown in Fig. 4e, the concentration of AA was significantly lower in LPS-stimulated shFABP4-transfected cells than in LPS-stimulated normal cells (21.09 ± 5.88 vs 91.39 ± 4.38 ng/mL; *t* = 17.81, *P* < 0.001). Collectively, these data suggest that silencing of FABP4 gene expression by RNA interference inhibited the AA–COX2 cascade.

## Discussion

Our previous work using genomic and proteomic approaches showed that FABP4 expression was upregulated in an animal model of PTE. In addition, FABP3 has been reported to be a promising indicator for PTE [18] and similar to FABP4 in structure [19], which suggests FABP4 could be closely related to PTE. Then, we also found that inflammatory cytokines, such as TNF-α, IL-1β, and IL-6, were more abundant in patients with PTE than in healthy subjects. Therefore, we hypothesized that FABP4 may be involved in the inflammatory response in PTE. Although several studies have demonstrated the importance of FABP4 for macrophage inflammatory responses, the specific contributions of FABP4 to inflammation in HPAECs has not been fully explored.

Here, we found that downregulation of FABP4 mRNA and protein levels reduced the inflammatory cytokines in HPAECs. We further investigated the link between FABP4 and inflammation in HPAECs. LPS treatment of inflammatory cells is known to stimulate the release of inflammatory cytokines [20, 21]. We found that silencing of FABP4 decreased inflammatory cytokine production in LPS-stimulated endothelial cells, which is consistent with previous reports of a relationship between FABP4 and inflammatory cytokine production in inflammatory cells [22–24]. While the mechanism by which this occurs had remained elusive, our research sheds light on this by showing FABP4-mediated modulation of the inflammatory response in HPAECs.

Our study provides the first evidence that FABP4 potentiates LPS-induced inflammatory response in HPAECs. Recent studies have shown that FABP4 modulates macrophage inflammatory responses through a positive feedback loop involving c-Jun kinase and activator protein-1 [25]. FABP4 contributes to lipid metabolism [26], and AA, a major long chain polyunsaturated fatty acid, has been reported to regulate fat metabolism [27, 28]. We showed here that LPS stimulates the expression of FABP4 in HPAECs. Interestingly, our finding that FABP4 deficiency reduced LPS-induced inflammatory cytokines, which are the downstream molecule of AA pathway, suggests that FABP4 may modulate the AA pathway during inflammation.

In this study, we found that FABP4 inhibition suppresses the LPS-induced AA pathway and downstream generation of inflammatory cytokines, such as TNF-α, IL-1β, and IL-6, in HPAECs. This result implies that FABP4 inhibitors could be used to prevent the development of inflammation-induced diseases.

Another finding in this study is that shRNA-mediated knockdown of FABP4 attenuates LPS-induced activation of the AA–COX2 cascade in HPAECs, suggesting that FABP4 is required for activation of AA signaling. Recent studies have shown that LPS stimulation induces AA release from the cell membrane [29, 30]. We showed here that FABP4 silencing significantly decreased AA release from membranes in the inflammatory status. Silencing of FABP4 prevented expression of COX2, which catalyzes the metabolism of AA to PGE2, a major lipid mediator involved in various inflammatory diseases [31]. Our results demonstrate that FABP4 expression was significantly associated with expression of an enzyme and metabolite of the AA pathway in HPAECs. However, the mechanism by which FABP4 activates the AA pathway is currently unknown.

This study shows that the potentiating effect of FABP4 on AA activation is dependent on its fatty acid-binding capacity. FABP4 is a peripheral protein with a high affinity binding site that non-covalently binds to fatty acids, increasing their solubility and transporting them from the lipid membrane to their sites of action [32–34]. AA and its metabolites may thus play a role as the primary or secondary signal in the signal transduction pathway [35]. We speculate that FABP4 is a ligand for AA [19] and plays a role in both signal transduction and in transport of fatty acids. In addition, FABP4 can bind to eicosanoids such as PGE2 and is involved in their metabolism and function [36]. Whether or not FABP4 potentiates activation of the AA signaling pathway through transport and transduce warrants further investigation. Our findings further support the notion that FABP4 lies at the crossroads of lipid metabolism and inflammation and also raise the possibility that activation of long chain fatty acids such as AA may also depend on FABP4 in HPAECs.

## Conclusions

We show here that FABP4 RNA interference inhibited the inflammatory response in HPAECs, and that FABP4 and AA metabolism are involved in perpetuating the inflammatory response. Furthermore, disruption of FABP4 activity attenuated the AA cascade and downregulated production of inflammatory cytokines. Taken together, our findings that FABP4 inhibition suppresses the inflammatory response in vitro and that inflammation is associated with thrombus formation [37–39] suggest that FABP4 plays a key role in PTE-associated inflammation and be associated with resistance to conventional diagnosis on inflammation-associated diseases.

## Abbreviations

AA: Arachidonic acid
COX2: Cyclooxygenase 2
FABP4: Fatty acid-binding protein 4
IL-1β: Interleukin-1 beta
IL-6: Interleukin-6
LPS: Lipopolysaccharide
PGE2: Prostaglandin E2
PTE: Pulmonary thromboembolism
shRNA: Small hairpin RNA
TNF-α: Tumor Necrosis Factor-α
